# Equitable Palliative care In the Community through Primary Care (EPIC-PC) study protocol: a realist study to propose a new integrated neighbourhood team approach to palliative care

**DOI:** 10.1136/bmjopen-2026-116327

**Published:** 2026-06-22

**Authors:** Sarah Mitchell, Jacqueline Birtwistle, Aaishah Javeed, Jude Beng, Lisa Hulbert, Carole A Paley, Emilie Couchman, Paola Cocco, Matthew Allsop, Justin Aunger, Edward Webb, Bryony Dawkins, Fliss E M Murtagh, Catherine Evans, Lucy Ziegler

**Affiliations:** 1 Academic Unit of Palliative Care, Leeds Institute of Health Science, Worsley Building, Clarendon Way, University of Leeds, Leeds, UK; 2 Academic Unit of Palliative Care, Leeds Institute of Health Science, Worsley Building, Clarendon Way, PPIE Co-Author, Leeds, UK; 3 Academic Unit of Health Economics, Leeds Institute of Health Sciences, Worsley Building, Clarendon Way, University of Leeds, Leeds, UK; 4 Department of Applied Health Sciences, University of Birmingham, Birmingham, UK; 5 Wolfson Palliative Care Research Centre, University of Hull, UK, Hull York Medical School, Hull, UK; 6 King’s College London, Bessemer Road, London, Cicely Saunders Institute of Palliative Care and Rehabilitation, London, England, UK

**Keywords:** Palliative Care, Terminal Care, Primary Health Care, Health inequities, Health Services

## Abstract

**Introduction:**

Avoidable and unfair variation in access to palliative care exists for different groups of people and communities. Primary and community care teams deliver most palliative care and care to people at the end of life at home but the quality of care provided is variable. This is an under-researched area and receives little attention in service design and policy. This study will investigate the key contexts, resources and components required for an integrated approach to palliative care to deliver improved and more equitable outcomes for patients and carers.

**Methods and analysis:**

This mixed-methods study adopts a realist methodological approach, and comprises four work packages:

A multi-perspective mixed-methods study to understand patient preferences and priorities in palliative care, prioritising recruitment of patients and family members/carers from areas of socioeconomic deprivation. Data collection will comprise: (1) qualitative interviews, (2) review of patient case notes and (3) a discrete choice experiment. Realist analysis will result in the development of theory based on the identification of the key contexts and underlying mechanisms required to achieve beneficial outcomes through an integrated approach to palliative care.

A realist evaluation of existing integrated models of palliative care will involve theory-refining interviews and theory-consolidating focus groups with professionals working in three different service areas.

Dynamic simulation modelling of the healthcare resources needed to deliver the proposed integrated approach, ensuring quality and equity.

The theoretical and economic modelling will be tested out at two expert stakeholder workshops to determine the key enablers to implementation in practice.

**Patient and public involvement:**

The study design was informed by patient and public involvement (PPI) with 16 patients and members of the public from diverse and socioeconomically deprived communities for 12 months in a National Institute for Health and Care Research-funded palliative care partnership. PPI will be continuous throughout the study, prioritising inclusivity.

**Ethics and dissemination:**

Ethical approval was obtained from the East of Scotland Research Ethics Service Research Ethics Committee 2, on 20 August 2025 (IRAS ID: 354755) and Health Research Authority approval on 1 October 2025. The targeted dissemination strategy will include outputs and resources for key audiences including patients and families, professionals in primary care and specialist palliative care and service commissioners. The results will inform service delivery to reduce inequities and optimise the use of finite resources to maximise impact.

**Trial registration details:**

The study is registered with the ISRCTN UK Clinical Study Registry: https://www.isrctn.com/ISRCTN61092011.

**STRENGTHS AND LIMITATIONS OF THIS STUDY**

- A specific strength of this study is that it will purposively recruit patients living in very socioeconomically deprived areas with palliative care and/or end-of-life care needs, and their family members or carers, who often have less opportunity to participate in research compared with patients in more affluent areas.
- A potential limitation is recruitment of patients through Deep End general practice, which are the practices that are most stretched in terms of funding, workforce and capacity for research.
- This is a health services research study, designed to inform the development of integrated palliative care services, in line with national policy in England which calls for a shift from hospital to community.
- The study design was informed by extensive patient and public involvement with people from diverse backgrounds, through a previous National Institute for Health and Care Research funded palliative care partnership and intends to address longstanding inequity in palliative care.
- A strength of this study is the use of realist methods, informed by a theoretical framework relevant to behaviour change and implementation in healthcare, to ensure that the research leads to implementable recommendations and patient benefit.

## Introduction

 Health inequity is unfair and avoidable variation in healthcare provision across the population. This is a major research and UK government policy priority. Health inequities are rising, with people in the most deprived areas living shorter lives and more years of their life with long-term conditions, advanced disease and ill health.^1^

Over 50% of people in England now die in the community, including in care homes,^2^ but emergency hospital admissions rise towards the end of a person’s life. Current policy advocates for a reduction in hospital admission for people at the end of life, given the economic cost and the fact it may not be in line with patient preferences. Contacts with out-of-hours urgent and emergency care services rise towards the end of a person’s life, and triggers for hospital admission are poorly understood.^3 4^ There is evidence to suggest that this is more likely for people from areas of socioeconomic deprivation, suggesting a deficit in support to enable care at the end of life at home.^5^

Palliative care is holistic, person-centred care, focused on quality of life for people with advanced disease and their carers. There is increasing evidence to support the need for, and cost-effectiveness of palliative care.^6^ Identification of palliative care needs, described as a ‘golden ticket’ to enhanced care in the community, is inconsistent,^7^ particularly for people with non-cancer conditions.^8^ People from areas of high socioeconomic deprivation, with non-malignant disease or from minority ethnic backgrounds, are less likely to receive specialist palliative care services compared with white British people, people from more affluent areas and those with cancer.^5 9^ Patients identified as having palliative care needs are more likely to be from affluent areas, compared with those in areas of socioeconomic deprivation.^10 11^

Primary care services, including general practice (GP), deliver most palliative care and care to people at the end of life in the community. A key characteristic of good community palliative care is continuity with primary care teams, which is associated with less frequent emergency healthcare use towards the end of life for patients of all ages.^812^,^14^ However, primary care approaches to the delivery of palliative care, and end-of-life care, are highly variable. Barriers include time pressures, compromised continuity, inconsistent training and variable integration with specialist palliative care services,^15^ and this is especially challenging in practices in socioeconomically deprived areas of England, where both funding and workforce are stretched. The ‘care of people who are terminally ill or suffering from long-term chronic disease’ is an essential service within the current National Health Service (NHS) GP contract, but over recent years, the palliative care register has attracted only three Quality and Outcomes Framework points (worth a total of around £640.00 per practice per year), and these were retired in April 2025.^16^ Overall, the elements of the GP contract related to palliative care and end-of-life care have changed little since 2007, the impact is under-researched and evidence to inform change is lacking.

With the introduction of the Health and Care Act 2022, and publication of the 10 Year Health Plan in 2025, health and social care systems in England are moving towards more integrated ways of working (Neighbourhood Health models), which aim to improve access, experience and health outcomes for patients and communities.^17^ A core function of these models of care is proactive identification of patients who could benefit from personalised care and continuity, both key characteristics of good community palliative care. However, the evidence to inform integrated models of palliative care within primary care is limited.^6^

Integration in health and care describes services working together so that individuals receive co-ordinated, personalised and seamless care. Effective integration occurs at interpersonal, team, organisational and system infrastructure levels.^18^ The term ‘neighbourhood’ describes a population of 30 000–50 000 people served by a network of GPs, working in partnership with other health and social care providers. Integrated Neighbourhood Health models may include co-located generalist and specialist multidisciplinary teams, community engagement and workforce development and training.^17^

### Review of existing evidence

To inform this research proposal, the team conducted a UK-focused rapid systematic review in December 2023 of empirical studies related to integrated primary and specialist palliative care services for patients with palliative care and/or end-of-life care needs. Studies relating to palliative care provided exclusively by specialist services in the community (including hospices) or in acute hospitals were excluded. Searches were carried out in MEDLINE, CINAHL, PsycINFO, CENTRAL and EMBASE databases, from inception to the present day, using broad search terms (‘Palliative Care’, ‘Integration’, ‘Primary Care’, ‘Inequalities’) modified and adapted for each database. Only five studies met the inclusion criteria, representing a heterogenous, low level evidence base to inform integration of primary and palliative care as follows: one case note study,^19^ one retrospective cohort study of patients with cancer primary care service use,^20^ one realist evaluation of an integrated care model,^21^ one single-blind randomised controlled trial of a short-term integrated palliative care service for patients with multimorbidity,^22^ and one mixed-methods study using ethnography, qualitative interviews and service user consultation.^23^ Four studies were limited to one site,^1921^,^23^ the cohort study included primary care data from the whole UK.^20^

The review findings are consistent with previous research (including the work of this research team). Overall, the studies revealed low rates of identification of palliative care needs for patients, especially where multimorbidities are present. Evidence suggests a lack of understanding about roles, responsibilities and what constitutes good community palliative care among professionals. The key findings across the five studies were:

- Care is poorly coordinated within primary care, especially in the last year of life.
- Prompt identification of the palliative care needs of patients can lead to timely assessment and care planning. Identification of patients with cancer who could benefit from palliative care is better than for those with non-malignant disease.
- Multimorbidity and the identification of palliative care needs are associated with increased primary care consultations, more prescriptions and referrals to specialist services. Older patients are less likely to have their palliative care needs identified.
- Integration between palliative care specialists, primary care and community nursing teams provides continuity and a means of managing or escalating difficult symptoms.
- Health record sharing is inconsistent and handover information from out-of-hours contacts may not always be helpful.
- There are inequities in access to palliative care for different societal groups and communities. The reasons for this are unclear.

Key issues that future integrated approaches in primary palliative care could potentially address include:

- Improving the identification of palliative care needs in primary care, which is associated with increased primary care input and continuity.
- Understanding and addressing triggers for hospital admission at the end of life, particularly for patients in areas of socioeconomic deprivation.
- Describing effective ways of integrated working to improve continuity of care, with more clarity around the roles and responsibilities of members of the primary care and specialist palliative care teams to address time and resource pressures in both services.
- Describing ways to address variation in skills, training and confidence, including in out-of-hours primary care, through new ways of working.^24^

### Rationale for research

The need to address inequities in palliative care through a personalised approach is a policy priority. Evidence to support Neighbourhood Health models of integrated care for patients with palliative care needs and those at the end of life is urgently needed. This study will investigate how, when, why and for whom new integrated approaches can improve patient experience and outcomes and deliver more equitable care. The proposed research addresses gaps in the current evidence base and will lead to service and policy-relevant recommendations.

### Aim

To propose the key contexts, resources and components required for an integrated approach to palliative care, to deliver beneficial outcomes and improve inequity.

### Objectives

The research objectives are:

1. To understand patient and carer experience, priorities and preferences regarding the provision of community palliative care, with a focus on areas of socioeconomic deprivation.
2. To understand the service development and training needs of primary care and specialist palliative care staff to enable delivery of integrated palliative care in the community.
3. To propose how, when, why and for whom the approach improves outcomes (programme theory).
4. To define the potential economic value and impact of the integrated approach.
5. To determine the key enablers, including commissioning and contracting processes, to enable implementation of the integrated approach in practice.

### Research design and theoretical framework

An integrated approach to palliative care, and care for people at the end of life, in the community is a complex intervention, with multiple components and interactions with contextual factors, including the active input of individuals, social infrastructures, available resources and organisational, economic, political and cultural factors.^25^ This study will therefore use realist evaluation methodology, suitable for the study of complex interventions. Realist methodology assumes that the same intervention will not work in the same way for everyone, everywhere. Realist evaluation allows the investigation of how and why an intervention achieves beneficial outcomes (or not), by understanding the mechanisms (hidden cognitive or affective responses of stakeholders, in certain circumstances).^26^

The proposed research draws on an initial programme theory for integration of primary care and palliative care, developed through the Reducing inEQUalities through integration of Primary and Palliative (RE-EQUIPP) Care Partnership (NIHR 135170), informed by previous realist research to understand effective collaboration in healthcare.^27^,^29^ The planned research will consider the core interactions, resources and refinements needed for effective integrated approaches to palliative care in different socioeconomic community contexts. A theoretical framework relevant to behaviour change and implementation in community settings, drawing on the Behaviour Change Wheel^30^ and the UK National Institute for Health and Care Research (NIHR) Medical Research Council complex intervention framework,^31^ will inform the realist data collection and analysis. The research will lead to a proposed model of integration of palliative and primary care services, comprising a set of discrete components and economic model, suitable for further testing and evaluation. Stakeholder workshops using the ‘integrated Promoting Action on Research Implementation in Health Services’ (i-PARIHS)^32^ framework will lead to the development of principles for implementation in practice.

### Programme theory

Previous research undertaken by members of this team generated understanding and insights into the integration of primary care and palliative care. Existing programme theory of service integration informed the data collection and analysis (30), with data analysis of the evidence related to primary care and palliative care leading to the description of contexts (C), mechanisms (M) and outcomes (O) and a series of explanatory Context-Mechanism-Outcome Configurations (CMOCs), and the programme theory outlined in Box 1.^33^

Box 1 **Programme theory for integration of primary care and palliative care from the Reducing inEQUalities through integration of Primary and Palliative Care Partnership**

- Patient and public members described cultural norms and racism in current services (C1) and associated power imbalances (M1) that hinder access (O1). This is proposed as a point of entry into integrated partnerships between primary care and specialist palliative care services as follows:
- Increased recognition of current inequalities across the health and social care system (C2) has the potential to inform shared vision and purpose (M2), mobilising new, innovative, cross-boundary integrated partnerships to provide more equitable palliative care (partnership entry/O2). Transparency and honesty about these factors amongst both frontline professionals and service leaders (C3), underpinned by self-awareness and reflection (M3.1) and leadership behaviours that enable change (M3.2), can foster learning and commitment to improving inequalities (partnership functioning/O3).
- In a healthcare system where there is competition for finite resource (C4), models of integrated primary and palliative care require trusted interpersonal relationships (M4) and clarity around roles and responsibilities between professionals (O4). Positive experiences for patients, families and professionals, delivered through well-coordinated, multidisciplinary care (C5), reaffirms efforts (M5) and builds confidence in the approach (partnership synergy/O5). Further enabling contexts may include co-location of professional teams, reliable record sharing and working towards a shared goal.
- This theory provides a range of contextual factors amenable to change to trigger mechanisms and lead to positive outcomes for patients, families, carers and professionals.

### Study setting and sampling

The study setting is the North East and Yorkshire region of England, comprising four Integrated Care Board areas (South Yorkshire, West Yorkshire, Humber and North Yorkshire and the North East and North Cumbria). The region includes rural and coastal areas as well as urban and suburban towns and cities. It is the region of England with the highest proportion of households (54.6%) in England who are deprived in at least one of the four dimensions of education, employment, health and housing. Around 10.8% of the population are from minority ethnic backgrounds (6.3% Asian, 1.6% black, 2.9% mixed/other). This ethnic diversity is representative of England except for the more densely populated West Midlands and London.^34^ These population demographics will inform the sampling frame for the recruitment of patients and professionals. People living in areas of socioeconomic deprivation face multiple intersecting characteristics of disadvantage, including gender, ethnicity and disability. These factors overlap and increase further people’s disadvantage in (1) accessing the care and support they need and (2) taking part in research.^35^

At the time of this proposal, there are three ‘Deep End’ primary care networks in the region, supported by NIHR Regional Research Delivery Networks, to increase research capacity in the most deprived areas through research active GP. Across the region, we have identified three sites for realist evaluation who are already providing palliative care in the community through integrated approaches, suitable for evaluation in this study, including one model of clinical service integration, one model of organisational service integration and one model of administrative, data and record integration.

## Methods and analysis

This 3-year study began in February 2025 (end date 31 January 2028). The study consists of four linked work packages (WP) as follows:

### WP 1: to understand patient and carer/family experiences and priorities for palliative care

A multiperspective, mixed-methods study, to develop theory, comprising qualitative interviews with patients and family members/carers, followed by a detailed case note review and a discrete choice experiment (DCE). This WP will address the research question: ‘What are the priorities and preferences of patients, family members and carers for the provision of community palliative care and care at the end of life, including those from areas of socioeconomic deprivation?’

#### WP 1.1: patient and family/carer experiences

Design: A sequential, exploratory study with qualitative semi-structured interviews (n=35–40) with patients identified as having palliative care needs in primary care. Where appropriate, and with the patient’s permission, their family members or carers will also be interviewed or invited to attend. Interviews will be followed by detailed patient case note review.

Sampling: Recruitment will take place through primary care, including via the Deep End research networks, across the North East and Yorkshire. Purposive sampling to select participants with relevant experiences and the potential to provide rich data relevant to the research question will take place through engagement with GPs, with a sampling frame informed by the population demographics outlined in the 2021 census. We will aim to recruit at least 50% of participants from areas of socioeconomic deprivation (n=20–22), with 10% of participants from black and minority ethnic populations (Asian (n=2–3), black (n=1) and other (n=2–3)). Recruitment will be via 10 GPs, including Deep End practices, geographically spread across the region. Each practice team will identify potential participants (n=3–5) through a targeted search of their patient records. We will aim to recruit enough patients and carers for 35–40 interviews, but we expect attrition due to participants fluctuating healthcare needs, with the possibility that they will be approaching the end of life and become too unwell to participate. Inclusion and exclusion criteria are outlined in table 1:

**Table 1:** Work package 1 inclusion and exclusion criteria

Inclusion	Adult patients (aged ≥18 years) with advanced serious illness, registered with a GP practice in the North East or Yorkshire, who either: Receive specialist palliative care services. Are aware of (ie, had discussions about) palliative care, including inclusion on a practice palliative care register. Carers, including family members, (aged ≥18 years) of an eligible adult, invited to take part in an interview by the patient participant.
Exclusion	Adults with advanced serious illness who are unable to participate in a conversational interview for any reason. Adults with advanced serious illness who are unable to provide informed consent for any reason related to their condition (eg, becoming too unwell or approaching the end of life). Children and young people aged <18 years. Carers and/or family members who have not been invited to take part by the patient participant.

GP, general practice.

Potential participants will be provided with participant information by their clinical team. Participant information materials have been developed in partnership with members of the study Patient and Family Advisory Board. With verbal consent, the clinical team will provide the potential participant’s details to the research team, so that further information can be provided and a time agreed for an interview. All personal information will be stored securely, separately from any study data.

#### Data collection

Each participant will take part in one interview, which will be deliberately open and conversational. Following consent, the interview will begin with building rapport and collection of basic demographic information. A realist topic guide (online supplemental file) will inform the interviews, comprising open questions to allow participants to speak freely about their experiences and perspectives, with a series of prompts to ensure that patient and carer priorities and preferred outcomes are captured, including how and when these are achieved (or not).^36^ This will include details of how, when and by whom their palliative care needs were identified, their understanding of this and any the impact on the care they subsequently received. Specific aspects of care include opportunities for care planning discussions and referral to specialist palliative services (or not). All interviews will be in person, online or on the telephone, depending on patient choice. Where interpreters are required, these will be interpreters who are employed or approved by the University of Leeds, subject to a confidentiality agreement.

Interviews will last 40–60 min, to cover key topics but minimise participant burden. Given the nature of the interview, it is expected that some may take longer. The interview plan has been designed carefully to ensure that the risks and burdens associated with taking part are minimal, which is particularly relevant because the subject areas discussed during interviews may include the end of life and dying. If any of the participants experience difficulties, such as tiredness or distress, the interview will be halted and time offered for debrief with signposting to relevant support services if appropriate.

#### Case note reviews

Detailed review of the GP patient records of the patient participants, with their consent (n=30–35 as carers’ notes will not be included) will be conducted with the intention of adding further, detailed understanding of care. This has the potential to reveal nuances that patients may not be aware of, such as the outcome of referrals to specialist services. Case note review has advantages over database studies as details in free-text entries can be reviewed as well as clinical coding. Data for a 12-month period will be extracted to a bespoke data extraction table, with headings informed by the interviews and previous research on factors relevant to integrated primary and palliative care such as the date and time of palliative care identification and clinical codes used; health professional type and setting for recording palliative care need. The impact of identification of palliative care needs will be explored, including any free-text information detailing communication and consultations (including the health professionals involved), care planning, multidisciplinary team involvement, prescribing, access to benefits and referrals (eg, to social prescribers or specialist palliative care).

#### WP 1.1 data analysis

The first stage of qualitative data analysis from the interviews and case notes reviews will be reflexive thematic analysis, focusing on the priorities and preferences of patients, family members and carers using NVivo for data management. Thematic analysis is a method for identifying, analysing and reporting meaningful patterns (themes) within data.^37^ Analysis will begin alongside data collection, with familiarisation, reflection and note-taking. Codes will be assigned to every item of data, then grouped into overarching conceptual categories and themes. Analysis will be led by the Senior Research Fellow, with emerging findings discussed at monthly research team meetings. A selection of transcripts will be reviewed and independently coded by second researchers (SM and LZ), to reduce the risk of lone researcher bias, allow for comparison and further development of codes and lead to in-depth themes.

Exploratory analysis of data from the case-note reviews will identify patterns of care, drawing on data from interviews related to priorities and preferences and examining case notes to understand more about the contexts in which these are achieved. Analytical methods will include documenting the proportion of consultations that a participant has had since identification of palliative care needs with individual professionals, to characterise longitudinal continuity of care using the Usual Provider of Care index.^38^ To explore differences in the timing and receipt of care following the identification of need, for each participant we will develop a patient experience map,^39^ to depict the chronology of palliative care need identification and any variations in the subsequent episodes of care (capturing professional involvement and setting where possible).

### WP 1.2: patient priorities and trade-offs in palliative care provision

#### Design

A Discrete Choice Experiment (DCE) is a survey method which will provide further information on the aspects of community palliative care delivery that matter most to patients and carers (contexts and outcomes), as identified in WP 1.1. It will quantify which characteristics of community palliative care patients prioritise the most, and what trade-offs people are willing to make between them. This will help show how care that is aligned with patients’ priorities can be delivered efficiently with limited resources. In line with the project’s realist approach, the analysis will have an emphasis on when, how and for whom preferences differ across different groups. In a DCE, participants make choices between two hypothetical options. Each option is characterised in terms of several attributes, which could, for example, include waiting time for a GP appointment and continuity of care, since seeing the same professional for every appointment may require a longer wait. Participants make a series of such choices, with the levels changing in each task, for example, time to get an appointment could vary between 1 day and 2 weeks, and continuity of care could range from always seeing the same GP, to sometimes seeing someone else, to seeing a different GP each time.

We will examine how preferences differ for participants, according to their socioeconomic status, as well as looking at other characteristics, such as age, gender or ethnicity. Statistical analysis of how participants’ choices change as the attribute levels vary then reveals what they prioritise, and the trade-offs they are willing to make. For example, it could provide information on how long participants are willing to wait or how frequently they prefer GP appointments to maintain continuity of care.

#### Survey development

It is best practice to develop attributes using qualitative methods.^40^ Findings from WP 1.1 will inform the development of potential attributes, each describing a feature of palliative care that is important to patients. A prioritisation exercise with the Patient and Family Advisory Board will follow, to select the final attributes and design a draft survey. Basic (optional) demographic information will be collected that will be analysed and reported at aggregate level, but no identifiable information. The draft survey instrument will be piloted with patient and public involvement (PPI) members to ensure it is understandable, with minimum participant burden.

Sampling: The final version of the survey will inform sample size calculations, but the aim is to recruit as large a sample as possible of both patients with palliative care needs and carers, to facilitate examining preference differences over several different groups. We anticipate recruiting up to 300 participants, based on previous studies and in line with typical DCE sample size.^41^

#### Data collection

The survey will comprise questions to understand participant demographics and choices between hypothetical service delivery options. The survey will be distributed via collaborative partnerships with national voluntary sector organisations for patients (of all ages), families and carers. In addition, recruitment will be via GP, including through the Deep End networks across England, to ensure that responses are gathered from groups who are often under-represented in DCE survey samples. Most recruitment will take place online, but there will be an option to complete a paper survey, distributed in GPs and by post, to guard against digital exclusion.

#### Data analysis

Data will be analysed using appropriate econometric techniques, to be defined when the final survey is developed, with an emphasis on incorporating heterogeneity, such as mixed logit and latent class. Patient and carer samples will be analysed separately, and the results compared. The findings will provide valuable new insights into patient and carer priorities, how these differ for different people in different contexts, how these may be achieved and the possible trade-offs that can be made in service design to direct finite resources most effectively in line with these priorities.

#### Realist analysis and outputs

WP 1 will lead to new insights and understanding of patient and carer experience and preference in relation to community palliative care and end-of-life service delivery through three lines of enquiry: (1) patient and family/carer interviews, (2) case note reviews and (3) the DCE. Each of these will be analysed separately, then synthesised by applying a realist logic, to identify descriptions of key beneficial outcomes from a patient and family/carer perspective, for whom these are important, how these are achieved (or not) (mechanisms) and when (contexts). Outcomes will not be predefined but will be derived from the data.

Data from each analysis will be extracted into a bespoke data extraction table, according to whether it provides insights into the contexts (C) in which the preferred patient and family outcomes (O) are achieved, and the underlying mechanisms (M). Outcomes will be considered at different system levels, from patient and family related outcomes to professional outcomes, organisational and policy-relevant outcomes. Explanatory CMOCs will link contexts and mechanisms to outcomes, providing new insights and understanding into the contexts in which specific mechanisms are triggered, leading to desirable outcomes. The process will include regular team discussion and techniques including identifying patterns in the data, retroduction (inductive reasoning to derive new theory from multiple observations) and deductive logic (testing ideas against existing theory). This stage of the realist analysis will be informed by the programme theory proposed from the REducing inEQUalities through Integration of Primary and Palliative Care (RE-EQUIPP Care) Partnership (Box 1), and the study theoretical framework. Realist methods for analysis have distinct advantages in that multiple sources of evidence can be brought together, to develop and propose CMOCs and programme theory for further refinement in WP 2.

### WP 2: To understand professional perspectives

This WP will follow realist evaluation methodology to investigate ‘what works, for whom, when and in what circumstances’ in integrated community palliative care across three existing models. Data collection will comprise (1) interviews (theory-refining) and (2) focus groups (theory consolidating) with key professional stakeholders involved in the delivery, design and commissioning of three services in England, each with different types and levels of integration. Methods will adhere to the Realist and Meta-narrative Evidence Syntheses II (RAMESES II) quality standards for realist evaluation.^26^

A series of realist research questions will be addressed:

- What are the circumstances (contexts) in which preferred patient/family outcomes in palliative care are achieved through integration of specialist and primary care services? (When and how?)
- What are the characteristics and behaviours of professionals involved? (How and whom?)
- What are the potential beneficial outcomes from the perspective of professionals? (What works and for whom? Are there any unintended consequences?)
- What are the specific considerations for providing palliative care and care at the end of life through integration in areas of socioeconomic deprivation?

#### Sampling

Data collection will take place across three geographically dispersed and socioeconomically deprived areas in the North East and Yorkshire region of England, where community palliative care is already delivered through different types of integration (clinical service integration, organisational integration and administrative integration). Professionals who have experience of delivering integrated palliative care will be purposively sampled through a ‘snowballing’ approach, where participants help researchers to identify other potential individuals with relevant experience. Inclusion criteria are broad, to enable participation from a range of practitioners from primary care and specialist palliative care services including allied healthcare professionals and care coordinators (table 2).

**Table 2:** Work package 2 inclusion and exclusion criteria

Inclusion criteria	Professionals from primary care, community services and relevant specialist teams who have experience of delivering palliative care through an integrated approach. Professionals who have experience of providing palliative care in areas of socioeconomic deprivation, through an integrated approach.
Exclusion criteria	This is a community-based study, and so excludes participants who work exclusively in hospital trusts.

Interview participants will be professionals with relevant experience from community services and primary care (n=24), and specialist palliative care (n=12). The final sampling strategy will be informed by the findings of WP 1 to ensure that any key professionals identified in patient/family interviews are represented. Focus groups will follow, to include any key professionals who are not represented in the interview sample, and commissioners and service managers for each service who can provide more specific insights into the service development requirements. To ensure that focus groups are manageable, and every participant can contribute, there will be up to 10 professionals at each.

#### Data collection

The two stages of data collection are (1) online or in-person interviews, with professionals with experience of palliative care in the community (theory-refining), followed by (2) three focus groups (theory-consolidating). A realist topic guide will inform the interviews, derived from the findings of WP 1. As part of this, the emerging programme theory will be presented for participants to provide views and perspectives.

Study information will be shared widely in each of the study sites, through email newsletters, social media and presentations from the research team. Potential participants will make an initial expression of interest directly to the researcher, following which they will be contacted by the research team with further information, to plan for an interview and provide online consent. Interviews will be in person, online or on the telephone, depending on the preference of the participant, at a time that is mutually agreed so that it does not interfere with clinical commitments. Each participant will take part in one interview. Voice recordings of telephone or audio call interviews will be made using encrypted devices.

#### Focus groups

A minimum of three realist focus groups will be held, to bring together participants with varied experiences, to test the CMOCs further and consolidate the programme theory.^42^ Realist topic guides will provide a framework for the focus groups. (64) During the focus group, the researchers will use the ‘teacher-learner’ cycle to present emerging theories to participants and ask, ‘is this how you thought X should work?’

#### Data analysis

First, realist data analysis will be conducted from interviews from each of the three sites individually, then together. Every section of data will be coded according to whether it is functioning as a context (C), mechanism (M) or outcome (O) by two researchers. The analysis will inform the further development of the CMOCs from WP 1, drawing on the theoretical framework for the study including the Behaviour Change Wheel concepts of capacity, capability and motivation, to focus on behavioural influences on practice which are potentially amenable to change. Explanatory CMOCs proposed in WP 1 will be refined and refuted through identifying patterns in the data. Researchers are integral to the qualitative research process, so an ongoing process of discussion and reflection on the team’s experiences, values, beliefs and understanding around the topic during data analysis, is essential.

#### Output

This package of work will lead to a series of refined and consolidated CMOCs presented as a programme theory. This theory will underpin the integrated approach and an explanatory logic model of integrated, equitable community palliative care at interpersonal, organisation and system levels.

### WP 3: To define the potential economic value and impact of the integrated approach

An early economic evaluation using a dynamic simulation modelling approach will evaluate the potential impact of the proposed integrated approach to palliative care on the healthcare resources needed to deliver it, quality of care and inequities. A dynamic simulation model allows the various ways patients can move through the healthcare system to be mapped out, for example, capturing differences in timing of referral, and who delivers palliative care to different groups. These pathways are constructed based on patient characteristics, health system resources and other external factors which could influence the ways patients access care. In this way, the interactions between patients and health system actors are captured and used to predict future parts of the pathway and subsequent outcomes.^43^

#### Methods

The early economic evaluation will comprise two phases. In the first phase, a dynamic simulation model will be developed based on best practice methods^44^ to reflect current provision of palliative care in the community and the associated care systems patients must navigate to access this care. This approach will capture variation in patient navigation through the system depending on their health and socio-demographic characteristics, interactions with professionals and the health and care implications of these interactions. Patient experience maps will be informed by data collected in WPs 1–2, capturing variation in experiences in deprived versus less deprived areas, depending on patient characteristics, validated with PPI and clinician input. The associated healthcare costs and resources in current provision of palliative care in the community will be estimated by linking patient experience information, such as number and type of healthcare contacts, with associated healthcare cost information from NHS unit cost of healthcare services data. Subsequent patient outcomes will be incorporated into the model using information from WPs 1–2 related to the types of services that participants receive, the beneficial outcomes they associate with each service, whether they receive specialist palliative care services and quality-of-life data sourced from targeted literature searches. Variation in outcomes, based on patient profiles (demographics and clinical information) and the subsequent pathway to receipt of palliative care, will be captured. In addition, carer spillover effects will be explored by linking patient events with carer quality of life values identified from the literature.

From our previous research, we anticipate that outcomes will be identified that relate to patient and carers (such as shared understanding that the emphasis of palliative care is placed on lessening suffering, trusted relationships leading to care planning conversations, effective pain management), as well as system-level outcomes (such as continuity of care and care in a preferred care setting). These will feed into the economic evaluation by linking them with quality-of-life data (utility values) from the literature to enable analysis of quality-adjusted life years (QALYs) gained because of the integrated palliative care approach. This will allow quantitative estimation of the magnitude of inequities in access to palliative care based on demographic and clinical characteristics of patients in current practice.

In the second phase, the initial model will be adapted to reflect the provision of palliative care in the community with the adoption of the new proposed integrated approach. Using information about current pathways to care and the impact of specific interactions along that pathway, we will model what is likely to happen to patients if the pathways are changed because of the new integrated approach and the resulting impact on health outcomes and healthcare costs. This part of the analysis will be informed by data and recommendations about the implementation of the integrated approach from the other WPs, but as the new approach has not yet been implemented, it will focus on identifying the potential economic value of the new approach based on current evidence and drivers of uncertainty around this impact to inform future research.

#### Analysis

The economic analysis will be guided by the recommendations of the National Institute for Health and Care Excellence methods guide for health economic evaluations^45^ and will follow current recommendations at the time of analysis. The primary outcome for the economic analysis will be the incremental cost-per-QALY gained with the new integrated approach compared with standard practice. Estimates of the costs and care effects associated with the new integrated approach to palliative care will be estimated and compared with those associated with current provision to provide an indication of the potential economic value of the proposed approach. Cost-effectiveness will be determined by estimating the incremental cost-effectiveness ratio and comparing this with the relevant cost-effectiveness threshold at the time of analysis. Given the early nature of the evaluation and limited data to inform costs and effects of the new approach, the analysis will focus on the potential scope and magnitude of likely impacts, that is, what is likely/unlikely to effectively level-up provision of palliative care.^46^

The primary analysis will include patient outcomes and quality of life only, and a secondary analysis will also explore the inclusion of impacts on carer quality of life (and costs). This will be supplemented with equity-informative cost-effectiveness analysis, where appropriate, in which costs and outcomes (Quality Adjusted Life Years (QALYs gained) will be estimated for different population groups and presented as a distribution.^47^ The equity of the distribution can then be compared with the cost-effectiveness result. This will be plotted on the health-equity impact plane which demonstrates any trade-offs that may present between the cost-effectiveness and equity of the new proposed approach. Disaggregated estimates of costs and outcomes for different groups will also be presented to demonstrate the extent to which different population groups are expected to benefit from the proposed approach. Uncertainty around cost-effectiveness estimates will be explored using sensitivity analyses, for example, to explore assumptions made in the analysis or the impact of alternative parameters from different sources.

#### Output

An economic evaluation of the change in healthcare resources required for the new equitable approach to integrated palliative and end of life care, and potential change in care outcomes, particularly in deprived areas and access limited groups, compared with current provision.

### WP 4: To develop key principles for future service design and evaluation

Two half-day expert stakeholder consultation events will be held to co-design a set of key principles to inform future service design and evaluation of the proposed approach for integrated community palliative care, and economic model. The principles will underpin recommendations for implementation in practice.

The i-PARIHS (integrated-Promoting Action on Research Implementation in Health Services) framework will inform the event design.^32^ Use of the framework allows consideration of the intended recipients of the intervention and identification of key barriers and enablers to implementation. Implementation strategies and future evaluation of the new approach, across different contexts and recipients, can then be considered.

During the workshop, the refined logic model and economic analysis of the integrated approach will be presented to stakeholders, including Patient and Public Involvement (PPI) members, commissioners and clinicians. Guided by the Mi-PARIHS (Mobilising the i-PARIHS) Facilitation Planning Tool and resources,^48^ workshops will begin with a brief definition of the problem to be addressed (inequitable palliative care), description of the innovation (the integrated approach and economic model) and the recipients (patients and family/carers and professionals for whom there are beneficial outcomes). Guided by the framework’s domains and questions, discussions aim to build consensus and increase understanding of enablers and barriers to implementation across different contexts, for recipients. Discussions will include consideration of unintended consequences and implementing the approach in areas of socioeconomic deprivation. Principles and recommendations will be agreed and captured in real time by a live scribe.

#### Outputs

A set of recommendations and guidance to accompany the new approach, presented as a visual resource, to be shared with policymakers, commissioners and clinicians.

### Patient and public involvement

This study is directly grounded in the perspectives of the population the study aims to benefit, through extensive Patient and Public Involvement (PPI), conducted during the RE-EQUIPP Care Partnership (NIHR135170), co-led by SM and CE. This was a 12-month research and PPI partnership during which research questions were developed around how current inequity in palliative care can be addressed. The need for new research to provide solutions and avoid ‘one size fits all’ services, which are inaccessible to people from minoritised communities, was clear. This proposal was ‘pitched’ and tested with a panel of PPI members at a PPI ‘Dragon’s Den’ event in November 2022.^49^

A PPI strategy has been developed for the study, including recruitment of 17 PPI members with diverse backgrounds and a range of experience to the Patient and Family Advisory Board. A study PPI lead has been appointed to ensure consistent and effective co-ordination of activities and processes, in line with the UK Standards for Public Involvement in Research.^50^ Anonymised equality monitoring and engagement surveys will be conducted to understand our diversity (or lack of), so that we can proactively seek to provide opportunities for new members from under-represented backgrounds.

The Patient and Family Advisory Board will meet every 4–6 months to:

- Advise on the development of accessible study information and building trusted community partnerships with people from diverse and deprived backgrounds to enhance recruitment and data collection.
- Input into the design of health economics elements of the study.
- Support interpretation of study findings with identification of key messages and priorities from data analysis.
- Participate in expert stakeholder workshops.
- Be actively involved in dissemination activities and consideration of pathways to impact, including the development of visual resources from the study and presentations to key audiences.

Training needs of the board members will be identified and addressed, and strategies to maximise inclusive and meaningful involvement reported as part of the study dissemination plan. Evaluation to drive improvements in PPI will include an impact log and be recorded in the Public Involvement in Research Impact Toolkit (PIRIT).^51^

## Ethics and dissemination

Ethical approval was obtained from the East of Scotland Research Ethics Service Research Ethics Committee 2, on 20 August 2025 (IRAS ID: 354755) and Health Research Authority approval on 1 October 2025.

The findings from this study will have immediate relevance to service delivery, design and policy in the UK and internationally. Collaborating with the Patient and Family Advisory Board, we will ensure that the study outputs are accessible. A systematic and targeted dissemination strategy will enable timely sharing of outputs throughout the study, with timely identification of key stakeholders through a range of established professional networks across policy, professional, practice and patient organisations. Key audiences are policymakers and system leaders with responsibility for the strategic planning of healthcare services within the NHS in England and the Department of Health and Social Care, because this is where our policy understanding and greatest opportunity for impact exists. Engagement with public audiences is also important and we will work with PPI members to produce outputs to increase public understanding about what can reasonably be expected (or demanded) from primary and community healthcare services and specialist palliative care services, to help them to effectively navigate the health and care system.

## Supplementary material

10.1136/bmjopen-2026-116327 online supplemental file 1
