# Supplementary material for: Equitable Palliative care In the Community through Primary Care (EPIC-PC) study protocol: a realist study to propose a new integrated neighbourhood team approach to palliative care
Source: BMJ Open. 2026 Jun 22;16(6):e116327. doi: 10.1136/bmjopen-2026-116327 (PMC13289175; doi:10.1136/bmjopen-2026-116327)
Supplement: online supplemental file 1 [file bmjopen-16-6-s001.docx]

**WP1.1 Patient + carer interview guide**

Face to face, online or telephone: With Patient +/- Carer

**Introduction**

**Consent**

- Patient
  - Confirm consent to participate (written or audio or electronic informed consent), assess capacity
  - Clarify limit to confidentiality
  - Double check they understand about the data extraction
  - Check they know to tell the researcher if they don’t want to answer a question
- Carer
  - Clarify patient has given permission for carer to be present/participate
  - Obtain written consent for carer if participating in the interview(s)

**Interview length**

- approximately 45 mins, researcher to keep checking participant/s ok to continue

**Information about the interview:**

“*In this interview, we are interested in your experiences of healthcare when you are living with a condition that can’t be cured / serious illness, in particular, palliative care. What we mean by palliative care is the support provided by health professionals to help improve the quality of life for patients and families facing serious illness. This type of care is about helping to relieve physical symptoms, as well as supporting them with emotional, social, and spiritual aspects of their lives.”*

“*Before I switch on the recorder I will ask some questions about you, these are optional, and you can say if you don’t want to answer them*.”

**Complete demographic form (**some of this information may be different or missing in the electronic health record on data extraction**).**

**Confirm consent to audio record and switch on audio recorder**

**Topic guide**

| Question | Prompts | Logic |
| --- | --- | --- |
| Can you tell me a little about yourself and your story? | Your condition  Which health professionals and services you see  Do you talk to health professionals in person, telephone | Introductory,  Getting started,  Providing context. |
|  |  |  |
| What matters most to you in terms of your healthcare | Who is involved?  What do they do that is most important to you? How? When do these things happen?  Do you receive any continuity of care?  (specific health professionals that you see regularly – such as a particular team or member of a clinical team)  Who with? | **General understanding** of palliative care |
| Which healthcare professionals have been most important to your care | When you need help who do you prefer to call?   - How easy is it to contact them? - What are the issues with contacting them? - What would make it better | **Preferences /priorities**  **Context?** |
| What kinds of problems do you need healthcare professionals to address? | How do you get in touch with them?  Are they helpful?  What would make it better for you? | **Context**  **Preferences** |
| How do you want your GP to communicate with you?  th | - Would you prefer to find out via a letter, text, phone call, or a face-to-face appointment? - What information would you want to be told about palliative care? | **Preferences /priorities** |
| If applicable:  How did you feel when you heard you had palliative care needs? | - What does this term mean to you? - Is it helpful? - Did it feel appropriate - considering your healthcare needs? - Did you experience any strong positive or negative feelings? | **Context**  Patient’s understanding of palliative care as it applies to them |
| What difference has it made to have your “palliative care” needs identified? | - What is helping? - What could be better? - What is the impact on - your:   - use of health services?   - physical symptoms (e.g. pain, fatigue)?   - mental health (e.g mood, anxiety)?   - finances?   - access to benefit payments?   - access to medicines?   - spiritual care?   - anything else? | **Looking for mechanisms + outcomes**  (Having needs identified and how that helps) |
| How have you found out about the healthcare services you use? | - How easy is it for you to find what you need from the different healthcare services? - Who supports you with this:   - Healthcare professionals?   - Family and friends? - What problems have you faced? - What have you learnt/worked out for yourself? - Have you needed to spend your own money (e.g. travel, parking, obtaining prescriptions, paid care) - What would make you more confident? | **Looking for mechanisms + outcomes**  **(How they navigate services;**  **Health professionals involved and how they help)** |
|  |  |  |
|  |  |  |
| Patients with palliative care needs can sometimes be identified through a computer search. How would you feel if your GP identified that you had palliative care needs this way? | - How do you think patients would respond to this? - How do you think it would it have helped you? | **Preferences /priorities** |
| Have you any other thoughts or reflections you would like to share? |  |  |

**WP1 Patient Debrief Script**

*Keep the WP1 PISs Information Sheet and the “Patient Contacts for Further Information and Support” sheet with you during this conversation.*

| 1. **Thank the participant**  Thank you very much for taking part in this research project. This study looks at the experiences of patients, and sometimes caregivers, when receiving services from primary care – like GP surgeries and teams – and other services, when living with a condition that cannot be cured. |
| --- |
| 2. **Ask about their interview experience**  How do you feel the interview went? How did you feel about taking part today? |
| **3. Check for distress and signpost to support**  Some of the topics we discussed today can be sensitive. Did any of the issues we talked about feel distressing for you? If you would like any additional support, you can contact one of the organisations listed on the “Patient Contacts for Further Information and Support” sheet, or you can talk to your GP or palliative care team if you have one. You might not feel you need support at this moment, but please keep the sheet in case you do in the future. |
| **4. Remind them about withdrawal rights**  You have the right to withdraw from this research. You can ask us to remove, change, or delete data we hold about you by contacting the study researcher, Jakki Birtwistle – her details are on the information sheet. In some cases, we might not be able to remove your data if this would stop us from completing the research. If that happens, we will explain why. Also, once analysis has begun, it may not be possible to withdraw your data. |
| **5. Let them know about the findings**  When the research is complete, we will send you a summary of the findings through the same channel we first used to contact you. If you would prefer not to receive this summary, we can note this on your consent form. |

**Notes about the development of the guide:**

**Plan:**

A realist topic guide, informed by themes identified from the existing evidence base, will be piloted with two members of the PPI panel ahead of the interviews. The topic guide will comprise open questions to allow participants to speak freely about their experiences and perspectives, with a series of prompts to ensure that patient and carer priorities preferred outcomes are captured, including how and when these can be achieved. This will include:

- how, when and by whom their palliative care needs were identified
- their understanding of this
- impact this has had on subsequent care from their primary care team.

Specific aspects of care will include care planning discussions:

- when
- who with

Aspects such as:

- access to benefits via the Special Rules (SR1 form)
- referral to specialist palliative services (or not)

**Literature referred to:**

Leach I, Mayland CR, Turner N, Mitchell S. Understanding patient views and experiences of the IDENTIfication of PALLiative care needs (IDENTI-PALL): a qualitative interview study. British Journal of General Practice. 2024;74(739):e88.

Mitchell S, Leach I, Turner N, Mayland CR. Understanding patient views and experiences of the IDENTIfication of PALLiative care needs (IDENTI-Pall): a qualitative interview study protocol. BMJ Open. 2022;12(6):e062500.

**Could also check these**

Gao W, Gulliford M, Morgan M, et al. Primary care service use by end-of-life cancer patients: a nationwide population-based cohort study in the United Kingdom. BMC Fam Pract. 2020;21(1):76.

Dalkin SM, Lhussier M, Philipson P, et al. Reducing inequalities in care for patients with non-malignant diseases: Insights from a realist evaluation of an integrated palliative care pathway. Palliat Med. 2016;30(7):690-7.

Mason B, Epiphaniou E, Nanton V, et al. Coordination of care for individuals with advanced progressive conditions: a multi-site ethnographic and serial interview study. Br J Gen Pract. 2013;63(613):e580-8.

Evans CJ, Bone AE, Yi D, et al. Community-based short-term integrated palliative and supportive care reduces symptom distress for older people with chronic noncancer conditions compared with usual care: A randomised controlled single-blind mixed method trial. Int J Nurs Stud. 2021;120:103978
