# Supplementary material for: Equitable Palliative care In the Community through Primary Care (EPIC-PC) study protocol: a realist study to propose a new integrated neighbourhood team approach to palliative care
Source: BMJ Open. 2026 Jun 22;16(6):e116327. doi: 10.1136/bmjopen-2026-116327 (PMC13289175; doi:10.1136/bmjopen-2026-116327)
Supplement: Supplementary file 2 [file bmjopen-2026-116327.reviewer_comments.pdf]

PEER REVIEW HISTORY

BMJ Open publishes all reviews undertaken for accepted manuscripts. Reviewers are asked to complete a checklist review form and are provided with free text boxes to elaborate on their assessment. These free text comments are reproduced below.

ARTICLE DETAILS

Title (Provisional)

Equitable Palliative care In the Community through Primary Care (EPIC-PC) Study Protocol: A realist study to propose a new integrated neighbourhood team approach to palliative care.

Authors

Mitchell, Sarah; Birtwistle, Jacqueline; Javeed, Aaishah; Beng , Jude; Hulbert, Lisa; Paley, Carole A; Couchman, Emilie; Cocco, Paola; Allsop, Matthew; Aunger, Justin; Webb, Edward; Dawkins, Bryony; Murtagh, Fliss EM; Evans, Catherine; Ziegler, Lucy

---

VERSION 1 - REVIEW

**Reviewer**	**1**
**Name**	**Vladimir, Poroch**
**Affiliation**	**University of Medicine and Pharmacy "Grigore T Popa" Iasi, Palliative Medicine**
**Date**	**23-Feb-2026**
**COI**	**None**

It is a valuable, very interesting study, based on a mix of methods, which addresses a real and important public health problem.

I congratulate the authors for the idea and for the very well-argued and clear manner in which they revealed the conclusions of this study.

One observation: if a few references could be inserted to official documents (if any) to national programs or government strategies in the health system that denote the concern for ensuring equitable palliative care in the community.

VERSION 1 - AUTHOR RESPONSE

Reviewer: 1

Dr. Poroch Vladimir, University of Medicine and Pharmacy "Grigore T Popa" Iasi

Comments to the Author:

It is a valuable, very interesting study, based on a mix of methods, which addresses a real and important public health problem.

I congratulate the authors for the idea and for the very well-argued and clear manner in which they revealed the conclusions of this study.

One observation: if a few references could be inserted to official documents (if any) to national programs or government strategies in the health system that denote the concern for ensuring equitable palliative care in the community.

Response: I have checked that national policy references are included and up to date (references 15 and 16)
